# Cognitive decline due to ectopic primary hyperparathyroidism

**DOI:** 10.1002/ccr3.1901

**Published:** 2018-11-05

**Authors:** Yoshito Nishimura, Akira Yamamoto, Masahiro Takahara, Fumio Otsuka

**Affiliations:** ^1^ Department of General Medicine, Dentistry and Pharmaceutical Sciences Okayama University Graduate School of Medicine Okayama Japan

**Keywords:** ^131^I‐MIBI, ectopic parathyroid adenoma, hypercalcemia, hyperparathyroidism

## Abstract

When you see a patient with cognitive dysfunction, hypercalcemia due to hyperparathyroidism is an important differential diagnosis. It is important to consider including chest computed tomography and ^131^I‐MIBI SPECT examinations in patients with possible hyperparathyroidism and normal thyroid ultrasound.

## 1 CASE

**Figure 1:**
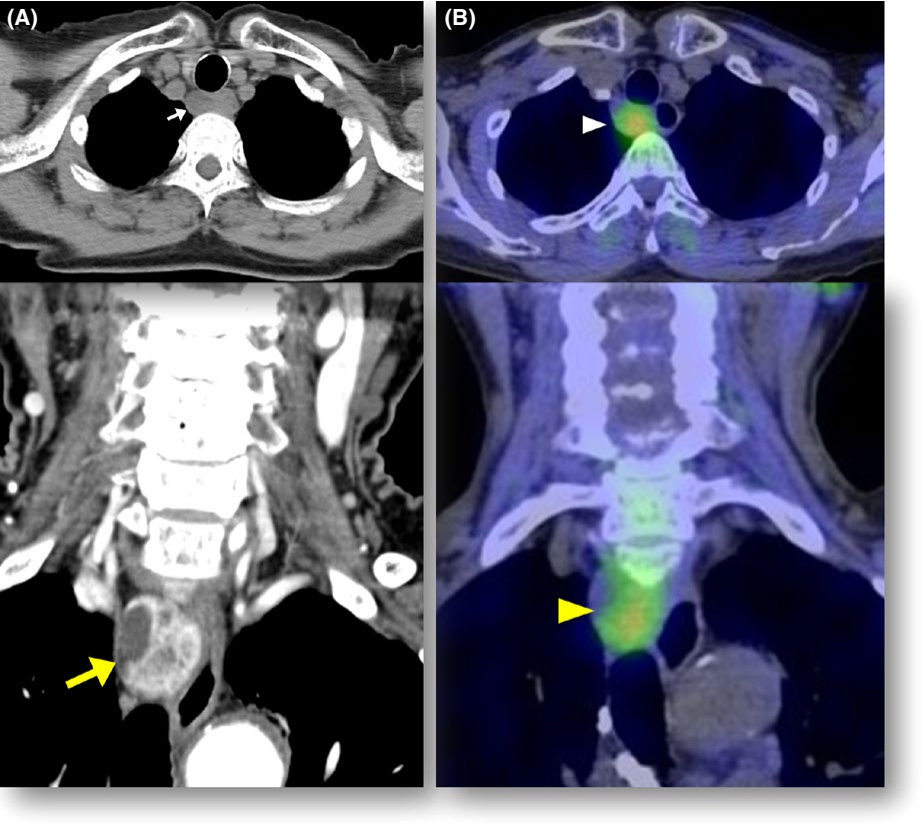
Horizontal view of noncontrast computed tomography (CT) of the mass (A, arrow). Contrast CT demonstrated enhancing mass measuring 2 cm in diameter in the posterior mediastinum (A, yellow arrow). ^131^I‐MIBI SPECT showed uptake in the mass (B, arrowheads)

A 77‐year‐old woman presented to our hospital with progressive cognitive decline. Laboratory tests were significant for corrected calcium of 12.3 mg/dL reference (range: 8.8‐10.1 mg/dL), phosphate of 2.4 mg/dL (range: 2.7‐4.6 mg/dL), and elevated intact parathyroid hormone of 549 pg/mL (range: 10‐65 pg/mL). Renal function was normal; a thyroid ultrasound demonstrated no tumors. Noncontrast computed tomography (CT) revealed a mass measuring 2 cm in diameter in the posterior mediastinum (Figure 1A, arrow). Contrast enhanced CT demonstrated a well‐defined enhancing mass (Figure 1A, yellow arrow). ^131^I‐methoxyisobutyl isonitrile (MIBI) single‐photon emission computed tomography SPECT) showed specific uptake in the mass (Figure 1B, arrowheads). Because her cognitive impairment was persistent despite the administration of calcitonin plus normal saline infusion, she underwent surgical resection of ectopic mediastinal parathyroid adenoma. Her symptoms improved postoperatively, and she reverted to eucalcemia.

Ectopic parathyroid adenoma causes approximately 6% of all hyperparathyroidism cases^1^, which is not uncommon. However, the diagnosis of ectopic parathyroid is often missed because of vague symptoms such as cognitive dysfunction due to hypercalcemia. It frequently occurs in the thymus and mediastinum.^2^ Because the sensitivity and specificity of identifying parathyroid adenoma with a single modality were low regardless of the technique,^2^ it should be considered to include chest CT and ^131^I‐MIBI SPECT examinations in patients with possible hyperparathyroidism and normal thyroid ultrasound.

## CONFLICT OF INTEREST

None declared.

## AUTHOR CONTRIBUTION

YN involved in literature search and drafting. AY involved in clinical care of the patient and revision. MT involved in clinical care of the patient and revision. FO involved in revision.
